# Supplementary material for: A New, Practical Animal Welfare Assessment for Dairy Farmers
Source: Animals (Basel). 2021 Mar 19;11(3):881. doi: 10.3390/ani11030881 (PMC8003747; doi:10.3390/ani11030881)
Supplement: Supplementary file 1 [file animals-11-00881-s001.zip › Full Welfare Monitor protocol.docx]

**Welfare Monitor protocol**

**Selecting animals for testing**

• A random sample can be obtained by selecting every n^th^ animal in the milking parlour. These animals are marked, to enable re-identification afterwards for data-collection.

• Alternatively, if animals can be locked in a feeding rack, they can be selected by choosing every n^th^ animal in the row(s). Data collection can be carried out immediately.

• In the least preferable method, animals in all areas of the pen including standing, feeding and lying animals are considered together.

• To simplify the assessment, animals can be marked with a stock marking device after

assessing them.
• The same animals can be assessed for the scoring of all measures, where random sampling is required.

• If animals are kept in different groups, proportionate sampling according to group size should be carried out.

• For all the measures that assess the quality of water provision the assessed pens are those in which the lactating animals are kept.

• Cleanliness of the body and integument alterations are assessed on the same side of each

animal.

**Number of animals needed for individual scoring**

| **Size of the herd** | **Number of animals to score** |
| --- | --- |
| 30 | 15 |
| 40 | 15 |
| 50 | 17 |
| 60 | 19 |
| 70 | 21 |
| 80 | 22 |
| 90 | 24 |
| 100 | 25 |
| 110 | 26 |
| 120 | 27 |
| 130 | 28 |
| 140 | 29 |
| 150 | 30 |
| 160 | 30 |
| 170 | 31 |
| 180 | 32 |
| 190 | 32 |
| 200 | 33 |
| 210 | 33 |
| 220 | 34 |
| 230 | 34 |
| 240 | 35 |
| 250 | 35 |
| 260 | 35 |
| 270 | 36 |
| 280 | 36 |
| 290 | 36 |
| 300 | 37 |

Table 1: Number of cows that have to be scored for the various individual tests

**1.Good feeding**

*1.1 Body condition score*

View the animal from behind and from the side in the loin and tail head area and assess the animals’ body condition. Animals must not be touched but only observed. Animals are scored as follows, with regard to 4 criteria and according to breed (see Figure 1). As yet, for the calculation of scores, only very lean animals (Sprecher score <1.5) are taken into account.

**Individual level:**

**0** – Regular body condition

**1** – Very lean: indicators for ‘very lean’ present in at least three body regions

**2** – Very fat: indicators for ‘very fat’ present in at least three body regions

Calculate the percentage of very lean cows (score 1)


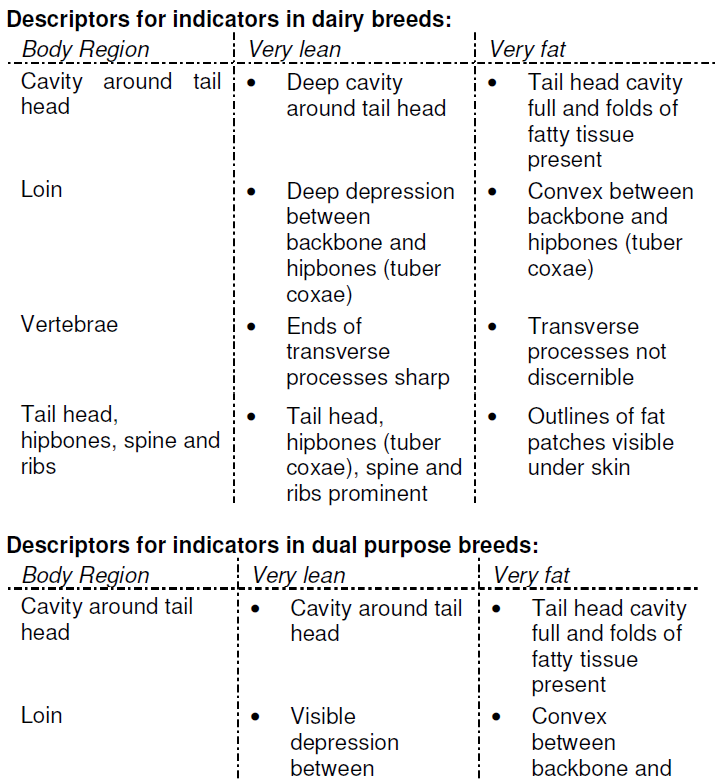


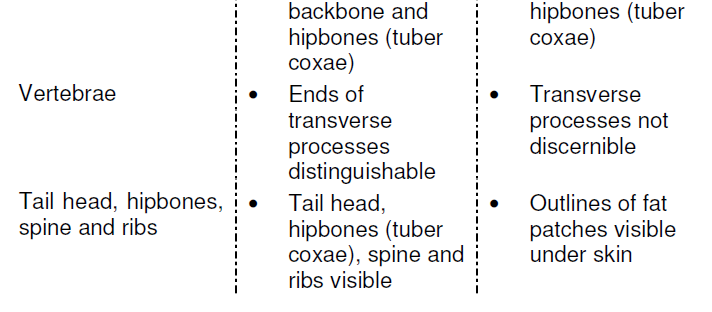


Table 2: Descriptors for body condition scoring.

Body condition score dairy breeds:

| 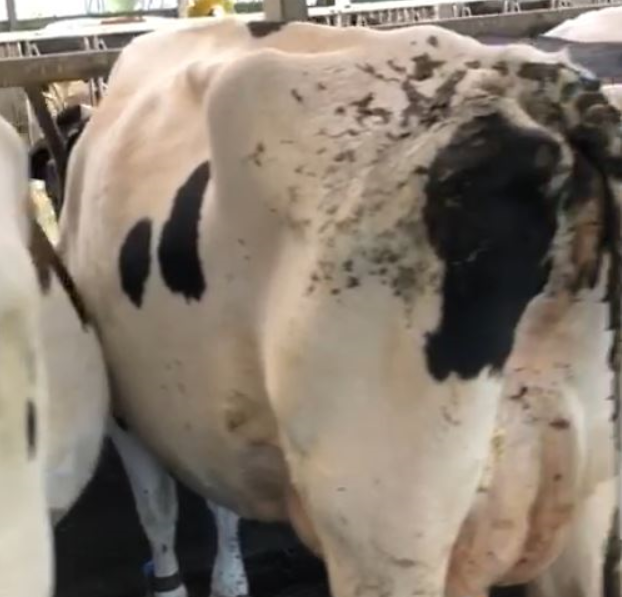 | 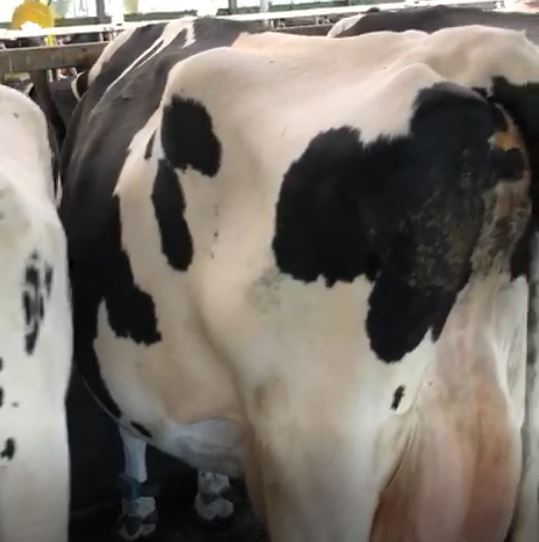 | 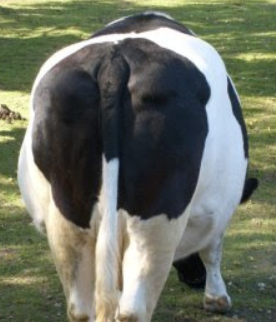 |
| --- | --- | --- |
| Score 1 | Score 0 | Score 2 |

Figure 1: Examples of body conditions. (After Welfare Quality protocol, 2009)

*1.2 Absence of prolonged thirst*

*1.2.1 Number of water points:*

All water points in question are assessed within the area of the animal unit where lactating animals are kept.
Count the number of animals per pen. In the case of open troughs, measure the length of the trough. In the case of bowls with reservoirs, bowls, nipple drinkers or drinkers with balls/antifrost devices, count the number of water points.

*1.2.2 Cleanliness of water points:*

All water points in question are assessed within the area of the animal unit where lactating animals are kept.

Check the cleanliness of the water points with regard to the presence of old or fresh dirt on the inner side of the bowl or trough as well as staining of the water. Water points are considered as clean when there is no evidence of crusts of dirt (e.g. faeces, mould) and/or decayed food residues. Note that some amount of fresh food is acceptable. A clean drinker scores 1, a partially dirty 2, and a dirty one 3 points. The average of all drinkers is computed.

Furthermore, check the amount of water coming out of the drinker per minute, e.g. by filling it up to the brim and then collecting the overflow for 1 minute using a bucket. To be sufficient the water flow must be at least 10 L/min in case of a bowl and 20 L/min in case of a trough. In the case of troughs with a large reservoir, this test does not have to be carried out. Water flow is then set to 20L/min. There should be at least 2 locations with drinkers within each pen.

2. Good Housing.

Two dimensions of the freestall are measured, the ‘barn environment’ as well as the softness of the bedding are also included.

*2.1. Dimensions of the cubicles*:
The width (distance between the dividers) and the diagonal (distance of the neck rail to the curb) of the freestall are measured. (Both measured as space between the tubing). Count the number of cows that are lying outside the stall, completely or partly (fig 2).


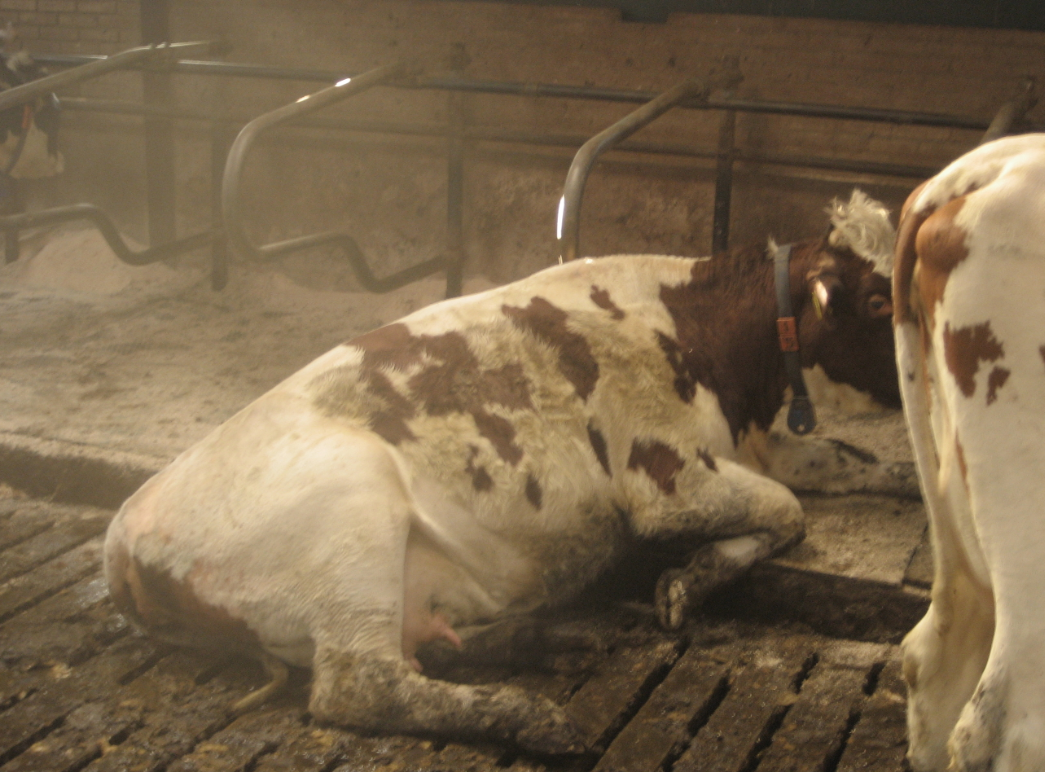


Figure 2: Cow that lies partly outside of the freestall.

*2.2. Cleanliness of the animals (Hygiene):*
The size of the dirty parts of the skin of the cows is measured during the clinical inspection (see 3.2. integument alterations). Measure the size of the dirty parts of the skin. Dirty parts smaller than 25x25cm are not considered relevant.

*2.3 Softness of the bedding*:
Softness of the bedding is measured with the knee test and can be classified as Good (soft), Moderately good or Insufficient (hard). For this test, one drops freely from a standing position on his/her knees, without touching anything, on the bedding. The level of pain experienced is the outcome. (cows do this 14 times per day)

*2.4 Barn environment:*
The barn environment is measured in three parameters: light, ventilation, and the presence of a mechanical brush.

Light: Good – everywhere in the barn it is easy to read a newspaper
 Partly – only at the feeding fence and some other places
 Insufficient – (almost) nowhere in the barn

Ventilation: Good – air in the barn smells fresh and ample options for ventilation
 Partly – air smells not so fresh and there are not many ventilation options
 Insufficient – air is dirty and few options for ventilation

Mechanical brush: Present or not

*2.4. Access to outdoor loafing area (OLA) or pasture:*

The animal unit manager is asked about the OLA and pasture management on the farm with regard to the availability of an OLA and/or access to pasture, and also the respective conditions in terms of days per year and average time spent per day in the OLA/pasture.

3. Good health

*3.1 Absence of injuries*

*3.1.1 Lameness*
Lameness describes an abnormality of movement and is most evident when the legs are in motion. It is caused by reduced ability to use one or more limbs in a normal manner. Lameness can vary in severity from reduced ability to inability to bear weight.

Indicators of lameness are:

• irregular foot fall

• uneven temporal rhythm between hoof beats

• weight not borne for equal time on each of the four feet

The following gait attributes are taken into account:

• timing of steps

• temporal rhythm

• weight-bearing on feet.

Assess the gait score of the animal. All animals should be walked in a straight line on a firm, level, non-slippery surface on which they would normally walk. The assessor should view them from the side and/or behind. Animals must not be assessed when they are turning.

Individual level:

0 – Not lame: timing of steps and weight-bearing equal on all four feet.

1 – Lame: imperfect temporal rhythm in stride creating a limp

2 – Severely lame: strong reluctance to bear weight on one limb, or more than one limb affected.

Calculate the % of animals with each lameness score

*3.2. Integument alterations (hairless patches and lesions/swellings)*

Integument alterations are defined as hairless patches and lesions/swellings. Only skin alterations of a minimum diameter of 2 cm at the largest extent are counted. Additionally, skin alterations in terms of hairless patches and lesions/swellings are counted in accordance

with criteria below. From a distance not exceeding 2 m, five body regions on one side of the focal animal have to be examined (fig. 3).

*Hairless patch* (see fig. 4):

• area with hair loss

• skin not damaged

• extensive thinning of the coat due to parasites

• hyperkeratosis possible

*Lesion/swelling* (see fig. 4):

• damaged skin either in form of a scab or a wound

• dermatitis due to ectoparasites

• completely or partly missing teats

• ear lesions due to torn off ear tags


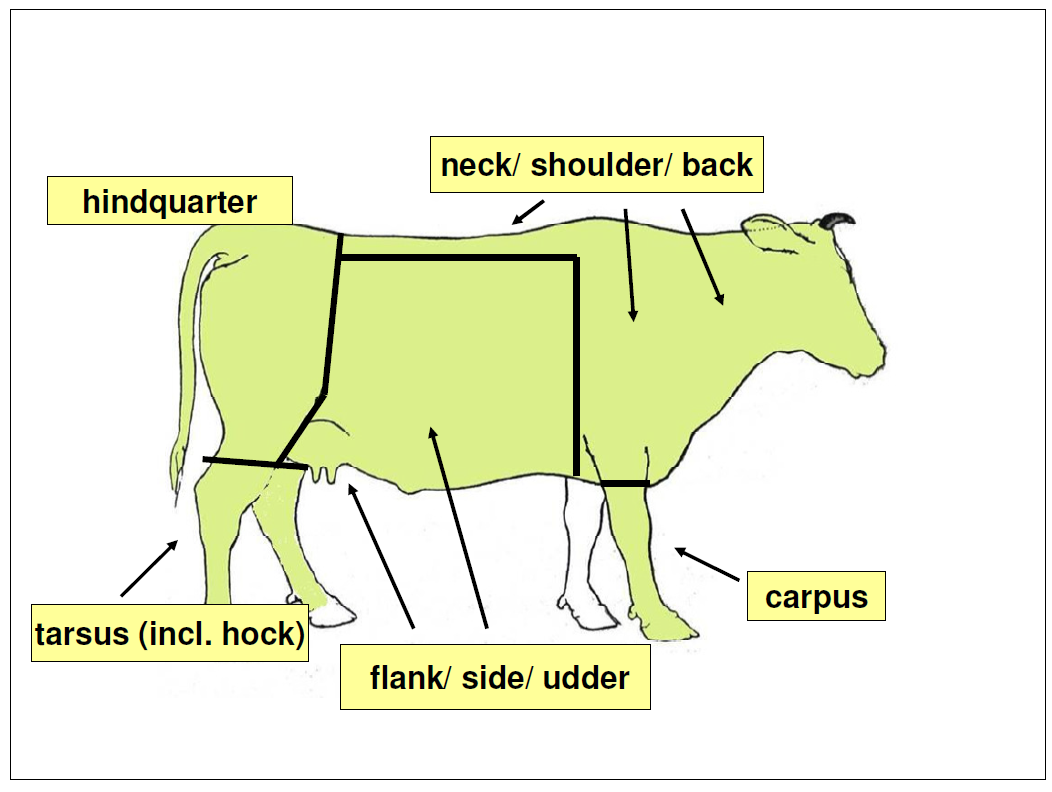

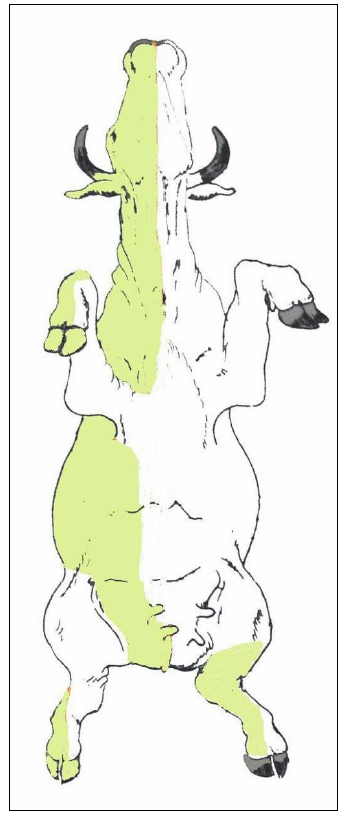


Figure 3: Regions to be examined for HP and lesions.(after Welfare Quality 2009).

| 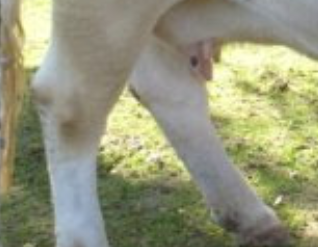 | 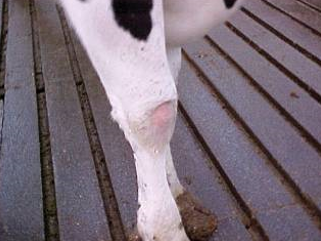 | 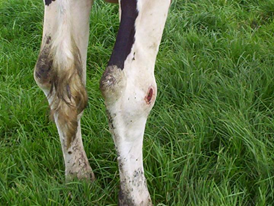 |
| --- | --- | --- |
| No hairless patches | Hairless patch | Lesion |

| 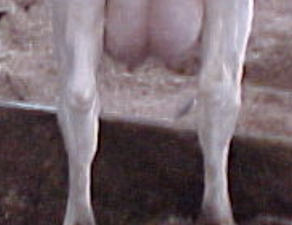 | 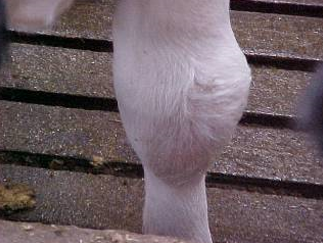 | 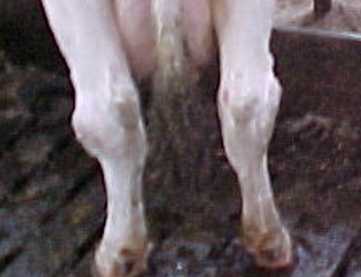 |
| --- | --- | --- |
| Normal hock joints | Swollen carpal joint | Swollen hock joints |

Figure 4: Examples of hairless patches and lesions.

*3.3 Absence of disease*
 *3.3.1 Coughing*Coughing is defined as a sudden and noisy expulsion of air from the lungs. It is recorded during the other observations/measurements. Total net observation time is 60 minutes.
Calculate mean number of coughs per animal and per 15 min.

*3.3.2 Nasal discharge*Nasal discharge is defined as clearly visible flow/discharge from the nostrils; transparent to yellow/green and often of thick consistency. The animal is observed but must not be touched. Animals are scored with regard to the nasal discharge criteria (see fig. 5).
Calculate the percentage of animals with nasal discharge.

| 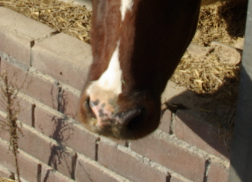 | 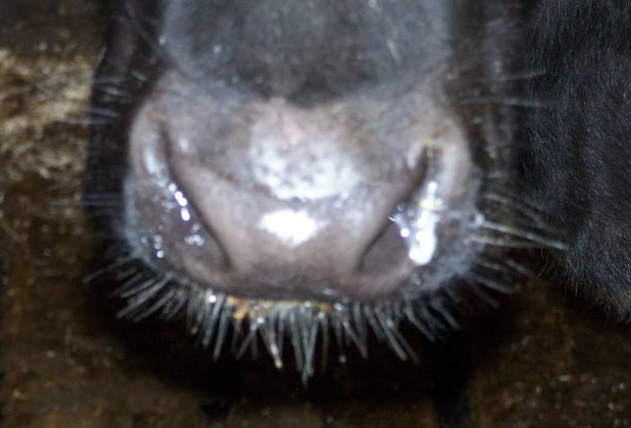 |
| --- | --- |
| No nasal discharge | Nasal discharge |

Figure 5: Nasal discharge

*3.3.3 Ocular discharge*Ocular discharge is defined as clearly visible flow/discharge (wet or dry) from the eye, at least 3 cm long. The animal is observed but must not be touched.
Calculate the percentage of animals with ocular discharge.

*3.3.4 Hampered respiration*
Hampered respiration rate is defined as deep and laboured or overtly difficult breathing. Expiration is supported by the muscles of the trunk, mostly accompanied by pronounced sound. Breathing rate may only slightly be increased. The animal is observed but must not be touched.
Calculate the percentage of animals with hampered respiration.

*3.3.5 Diarrhoea*Diarrhoea is defined as loose watery manure below the tail head on both sides of the tail, area affected at least the size of a hand. The animal is observed but must not be touched.
Calculate the percentage of animals with diarrhea.

*3.3.6 Vulvar discharge*
Vulvar discharge is defined as purulent effluent from the vulva or plaques of pus on the bottom side of the tail (CAVE: viscous mucus in animals in late pregnancy). The animal is observed but must not be touched.
Calculate the percentage of animals with vulvar discharge

*3.3.7 Somatic cell count*This measure requires input from animal unit manager. Milk somatic cell count data can be obtained from milk records. They are collected at individual cow level from a period of three months prior to the farm visit. Such data can also be collected in advance of the farm visit. Somatic cell counts greater than 400,000 are considered to indicate subclinical inflammation. Calculate the percentage of animals with somatic cell counts of 400,000 cells or above.

*3.3.8 Mortality*Mortality is defined as the ‘uncontrolled’ death of animals as well as cases of euthanasia and emergency slaughter. The animal unit manager is asked about the number of dairy cows which died on the farm, were euthanized due to disease or accidents or were emergency slaughtered during the last 12 months. Additionally the average number of dairy cows in the animal unit is asked. Farm records may also be used.
Calculate the percentage of animals that died, were euthanized or emergency slaughtered on the farm during the last 12 months

*3.3.9 Dystocia*

Dystocia incidence is defined as the number of calvings where major assistance was required during the last 12 months. Data is collected from herd records, or the animal unit manager is asked about the number of dystocia cases on the farm during the last 12 months (animal unit manager estimates). The average number of calvings (on a yearly basis) is also recorded. Calculate the percentage of animals with dystocia in a year.

*3.3.10 Downer cows*

Incidence of downer cows is defined as the number of cases of nonambulatory cows during the last 12 months. Data is collected from herd records, or the animal unit manager is asked about the number of downer cows on the farm during the last 12 months (animal unit manager estimates). The average number of dairy cows (on a yearly basis) is also recorded. Calculate the percentage of downer cows in a year.

*3.3.11 Disbudding/dehorning*

The animal unit manager is asked about the disbudding/dehorning practices on the farm with regard to the following items:

• Procedures used for disbudding of calves/dehorning of cattle

• Use of anaesthetics

• Use of analgesics

**0** – No dehorning or disbudding

**1** – Disbudding of calves using thermocautery/surgery

**2** – Disbudding of calves using caustic paste

**3** – Dehorning of adult cattle

and

**0** – Use of anaesthetics

**2** – No use of anaesthetics

and

**0** – Use of analgesics
**2** – No use of analgesics

*3.3.12 Tail docking*The animal unit manager is asked about mutilation management on the

farm with regard to the following items:

• Procedures for tail docking

• Use of anaesthetics
• Use of analgesics

**0** – No tail docking

**1** – Tail docking using rubber rings

**2** – Tail docking using surgery

and

**0** – Use of anaesthetics

**2** – No use of anaesthetics

and

**0** – Use of analgesics
**2** – No use of analgesics

**4. Appropriate behaviour**

*4.1. Avoidance test*
The test can start, when at least 75 % of the cows are back in the barn after milking. Place yourself on the feed bunk at a distance of 2 m (if possible) in front of the animal to be tested. The head of the animal has to be completely past the feeding rack / neck rail over the feed. Make sure that the animal is attentive or making notice of your presence. If an animal is not obviously attentive, but also not clearly distracted, it can be tested. A way to attract the animals’ attention is to make some movements in front of them (at the starting position). If you do not have 2 m in front of the animals for approaching them, then choose an angle of up to 45° with the feeding rack, and start at a distance of 2.5 m. If a distance of 2.5 meters is not possible, still carry out the assessment but note down the maximum distance possible on the recording sheet. Approach the animal at a speed of one step per second and a step length of approximately 60 cm with the arm held overhand in an angle of approximately 45° from the body. When approaching, direct the back of the hand toward the animal. Do not look into the animal’s eyes but look at the muzzle. Continue to walk towards the animal until signs of withdrawal or until touching the nose/muzzle. Definition of withdrawal is when the animal moves back, turns the head to the side, or pulls back the head trying to get out of the feeding rack; head shaking can also be found. In the case of withdrawal the avoidance distance is estimated (= distance between the hand and the muzzle at the moment of withdrawal) with a resolution of 10 cm (200 cm to 10 cm possible). If withdrawal takes place at a distance lower than 10 cm, the test result is still 10 cm. If you can touch the nose muzzle, an avoidance distance of 0 cm is recorded. Make sure that the hand is always closest to the animal during the approach (not the knee or the feet). Especially when getting close to animals that are feeding or have their heads in a low position, bend a little in order to try to touch them. Neighbouring animals that react to an animal being tested should be tested later on. In order to reduce the risk of influencing the neighbour’s test result, every second animal can be chosen. Retest animals at a later time if the reaction was unclear.

Record at Individual level:

Distance in cm (200-0 cm, with a resolution of 10 cm)

In the result of this test, the cows are grouped into 4 groups: 0 cm (can be touched); 0-50 cm; 50-100 cm; >100 cm.

*4.2. expression of normal behaviour*This is the number of days per year and hours per day with access to OLA/pasture as measured in 2.4.

**Calculation of scores for dairy cows on farm**

***1. Food and water***

*1.1 Absence of prolonged hunger*

The score of a farm in regard to absence of hunger is calculated from the % of very lean cows (that is with a body condition score of 1). This % is turned into a score using an I-spline function as follows: Let I = 100 - % of very lean cows,

A spline function is used to compute the index into a score, with the general formula:

Score F = a + b x I + c x I^2^ + d x I^3^

with a, b, c, d differing when I is lower or equal to a specific value (called knot) vs. equal or higher than this value.

The values for the knot and a, b, c, d are:

| knot | 80 |
| --- | --- |
| a when I < knot | 0 |
| a when I > knot | -2961.31541245367 |
| b when I < knot | 0.221659625378516 |
| b when I > knot | 111.270985374177 |
| c when I < knot | -0.00277074531758109 |
| c when I > knot | -1.39088729183631 |
| d when I < knot | 0.0000592709460062145 |
| d when I > knot | 0.00584308979366635 |

*1.2. Absence of prolonged thirst*

For each group of animals three aspects are considered:

• Is the number of functioning drinkers sufficient?

• Are there at least 2 drinkers available for an animal?

• Are the drinkers clean?

To be sufficient, there must be at least 1 water bowl for 10 cows and/or 6 cm of trough per cow. To be considered as partly sufficient, there must be at least 1 water bowl for 15 cows and/or 4 cm of trough per cow. A drinker that does not function properly counts for half. If a drinker is not functioning properly or the water flow is insufficient (i.e. lower than 20L/min for a trough or lower than 10 L/min for a bowl) then the recommended number of animals is divided by two (i.e. 1 bowl for 5 animals and 12 cm of trough per animal to be sufficient, and 1 bowl for 7.5 animals and 8 cm of trough per animal to be partly sufficient). After giving the score for the rest of the drinking related parameters measured, the score is divided by the average for the cleanliness (see fig. 6).

The score for absence of prolonged thirst is attributed to the group of cows according to the answers of the three questions as follows:


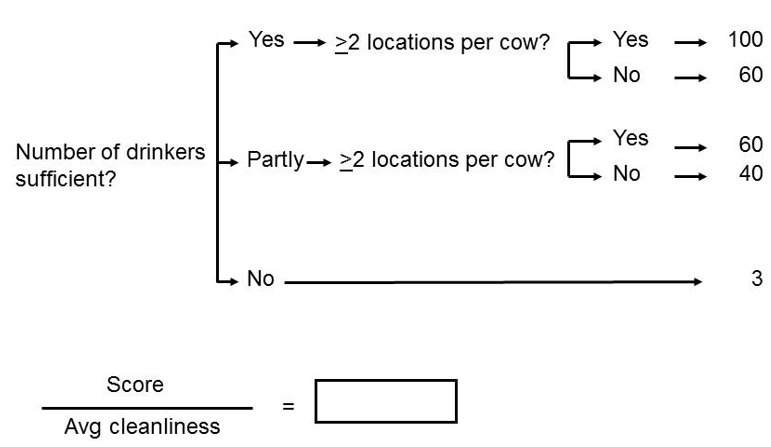


Figure 6: calculation of the score T for absence of thirst.


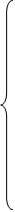


Principle score for good feeding = F+ (T-F)µ_2_ if F ≤ T
T+ (F-T)µ_1_ if T < F
μ_1_ = 0.12 μ_2_ = 0.27

*2. Housing*

*2.1. Dimensions of the cubicles*:

- If Diagonal ≤ 185cm = 9 points; else if 185cm < Diagonal < 195cm = 4 points; else = 0 points
- If Width ≤ 110cm = 9 points ; else if 110cm < Width < 120cm = 4 points; else = 0 points
- If % cows lying outside the stall ≥ 2% = 9 points; else if 2% > % Lying outside the stall ≥ 0% = 4 points; else = 0 points

These 3 scores need to be multiplied with 3 and summed to calculate A

*2.2. Cleanliness of the animals (Hygiene):*
The number of points belonging to the percentage of cows is presented in table 2. The sum of the points is the score for hygiene (H). If 15 > H ≥ 9 then B = 0 points; if 9 > H ≥ 7 then B = 4 points; else B = 9 points.

| Size of the dirty patch | 1 | 2 | 3 | 4 | 5 | points |
| --- | --- | --- | --- | --- | --- | --- |
| 25x25 – 50x50cm | >3 | >2 | >1 | >0.5 | < 0.5 |  |
| 50x50cm – ½ Hind Quarter | >1.0 | >0.5 | >0.25 | >0.15 | <0.15 |  |
| > ½ Hind Quarter | >0.5 | >0.25 | >0.15 | >0.1 | <0.1 |  |

Table 3: scoring for dirtiness of the skin. The percentage of cows having each category of dirty patch size is calculated and marked with 1 – 5 points. These are summed. Example: 1.5% of the cows had a dirty patch size 25x25 – 50 x 50 cm; 0.6% had a dirty patch 50 x 50 cm – ½ hind quarter and 0.3% was dirty > ½ hind quarter. This will result in 3 + 2 + 2 = 7 points. The score for hygiene H = 7 and B = 4.

*2.3 Softness of the bedding*:
Softness of the bedding is measured with the knee test and can be classified as Good (soft), Moderately good or Insufficient (hard). When it is Good: C = 0; Moderately good: C = 4; Insufficient: C = 9

*2.4 Barn environment:*
The flow chart for calculation of the score for barn environment (D) is presented in figure 7.

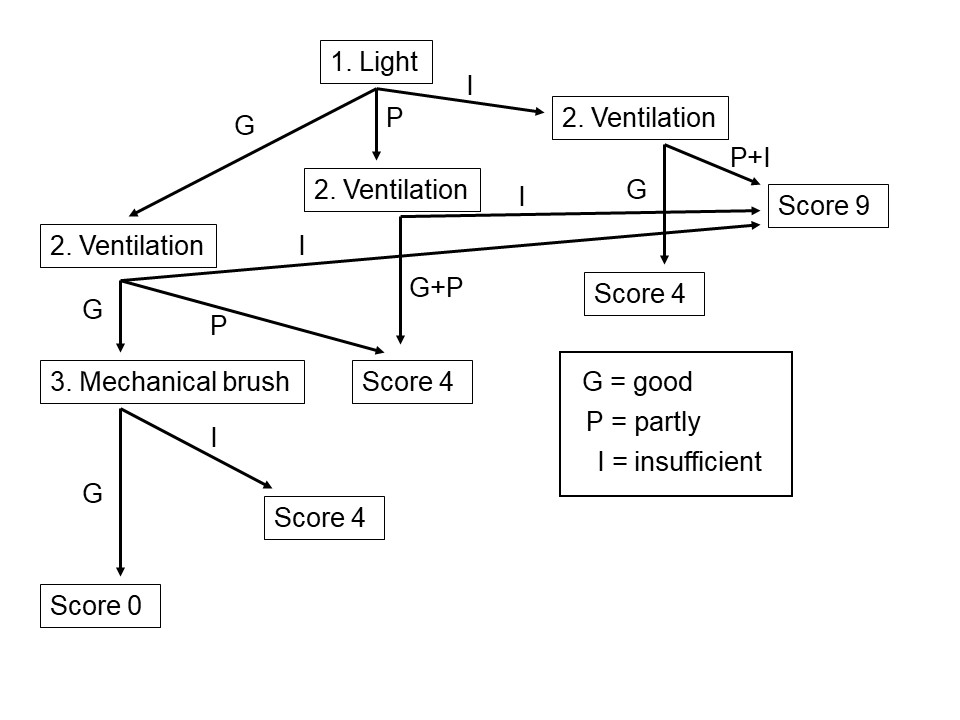

Figure 7: Flowchart for the scoring of the barn environment. few options for ventilation.

Finally, the index for comfort around resting (R) is calculated:

R=100-100*(A+B+C+D)/108
The sum is divided by 108 because of the theoretical maximum of the sum.

Score for good housing = R
(Because there is no assessment for thermal comfort yet and the welfare monitor is for freestall housing systems only, the score for good housing is only the comfort around resting)

***3. Health***

***3.1 Absence of injuries***

*3.1.1 Partial score for Integument alterations*

The average number of HPs, lesions and swellings per cow in the group is used in the calculations. Because a lesion or swelling is a more severe impairment for the welfare of the cow, it receives more weight in the calculations.

The Index for integument alterations is calculated as:

(2HP+5(lesions+swellings))x10
I(s) = 100 - 5

If I(s) ≤ 65 the score S becomes: (0.43 x I(s)) + (0.0065 x I(s)^2^) + (0.00013 x I(s)^3^)
If I(s) > 65 the score S becomes: 29.9 – (0.94 x I(s)) + (0.015 x I(s)^2^) + (0.00002 x I(s)^3^)

Where HP is the average number of HP’s per cow and lesions + swellings are also the average number of lesions and swellings per cow.

*3.1.2 Partial score for lameness*

The % of animals moderately lame and the % of animals severely lame are combined in a weighted sum, with a weight of 2 for moderate lameness and 7 for severe lameness (note that for tied cows only the proportion of severely lame animals is used). This sum is then transformed into an index that varies from 0 to 100 as follows:

2(% moderate) + 7(% severe)

Index for lameness I(l) = 100 - 7

A spline function is used to compute the index into a score, with the general formula:

Score **L** = a + b x I(l) + c x I(l)^2^ + d x I(l)^3^

with a, b, c, d differing when I(l) is lower or equal to a specific value (called knot) vs. equal or higher that this value.

The values for a, b, c, d and the knot are:

| knot | 78 |
| --- | --- |
| a when I < knot | 0 |
| a when I > knot | -2060.59837226844 |
| b when I < knot | 0.0987993246960187 |
| b when I > knot | 79.352519518838 |
| c when I < knot | -0.000954971884859655 |
| c when I > knot | -1.01702585933252 |
| d when I < knot | 0.0000534439143246927 |
| d when I > knot | 0.00439560501338991 |


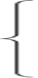
The two partial scores are combined using a Choquet integral. The parameters of the Choquet integral are: μs=0.56 and μl=0.31
 S+ (L-S)µ_4_ if S ≤ L
Partial score for absence of injuries = L+ (S-L)µ_3_ if L < S μ_3_ = 0.56 μ_4_ = 0.31
***3.2 Absence of disease***

Some diseases affect few animals in a herd while some other can spread very easily between animals. The incidence of symptoms of disease is compared to warning and alarm thresholds. The alarm threshold is the minimum value for a decision to put in place a health plan at the farm level. The warning threshold is half of the alarm threshold. The values chosen for alarm thresholds appear in Table 4.

The number of warnings and alarms obtained by a farm is calculated. At that stage, nasal and ocular discharges are considered together (ORL area) and coughing and hampered respiration are considered together (respiratory problems). If an alarm exists for one of the two symptoms of the same area then an alarm is attributed to this area. If a warning exists and no alarm, a warning is attributed to the area. Then the maximum of alarms and warnings is 8, equal to the number of distinct areas (ORL, respiratory problems, diarrhoea, mastitis, vulva discharge, dystocia, downer cows, mortality).

*Table 4 Warning and alarm thresholds for each symptom.*


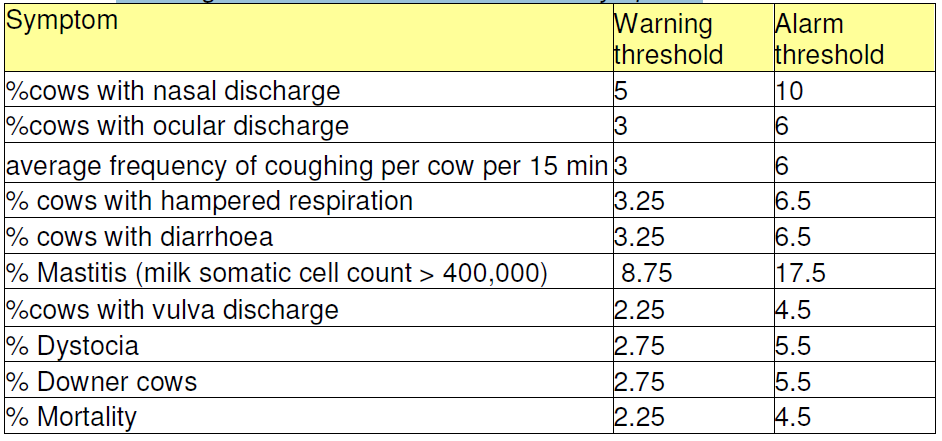


We calculate a weighted sum of warnings and alarms, with 1 the weight of warnings and 3 the weight of alarms. The theoretical maximum of this sum is 3 x 10 = 30.
To obtain an index (Id) between 0 and 100 (with 0-worst; 100—best), the sum is divided by the theoretical maximum and multiplied by 100 and the difference to 100 is calculated:
 I(d) = 100 – 100 x (sum/30)
A spline function is used to compute the index into a score, with the formula:

Partial score for absence of disease D = a + b x I(d) + c x I(d)^2^ + d x I(d)^3^

with a, b, c, d differing when I(d) is lower or equal to a specific value (called knot) vs. equal or higher than this value.

The values for a, b, c, d and the knot are:

| knot | 65 |
| --- | --- |
| a when I < knot | 0 |
| a when I > knot | -150.97697679629 |
| b when I < knot | 0.550170003688435 |
| b when I > knot | 7.49967223855853 |
| c when I < knot | -0.00478156849205967 |
| c when I > knot | -0.111009510561344 |
| d when I < knot | 0.0000725090260228106 |
| d when I > knot | 0.000611104858556935 |

***3.3*** ***Absence of pain induced by management procedures***

One score is attributed to dehorning and one to tail docking. These partial scores are attributed according to decision trees (Figure 8 and 9). Then at criterion level, the worst score among the two partial scores (one for dehorning and one for tail docking) is retained.

A farm is considered as practicing dehorning or disbudding when at least 15% of the animals present on the farm are dehorned or disbudded. The same principle is applied to tail docking.


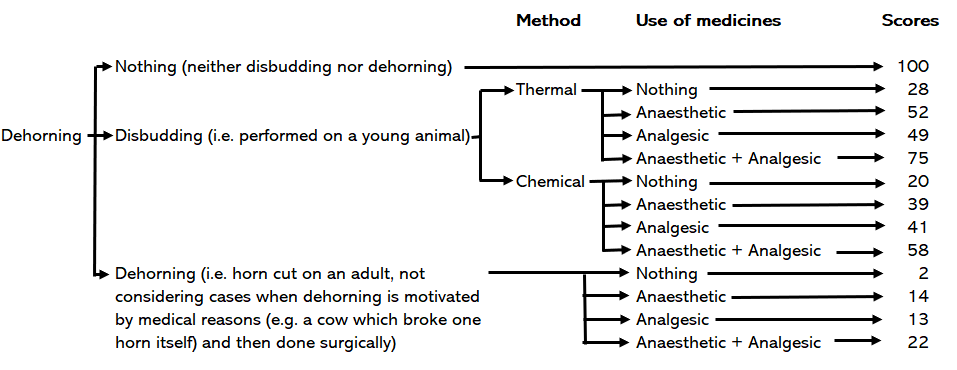


Figure 8: Scores attributed to combinations of answers to questions on dehorning (when surgical, follow thermal).


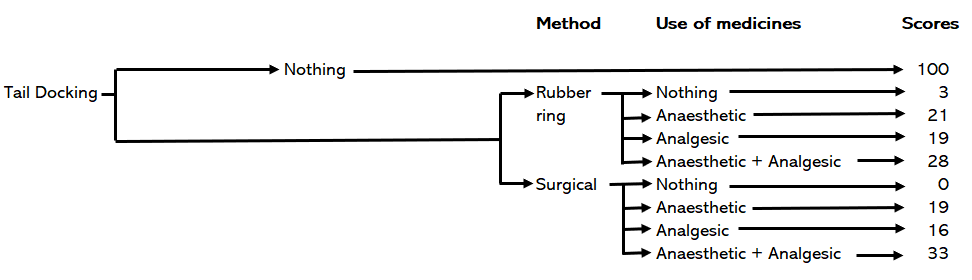


Figure 9: Scores attributed to combinations of answers to questions on tail docking.

This results in the calculation for the principle of Good Health:

H_1_ = partial score for absence of injuries.
H_2_ = partial score for absence of disease
H_3_ = Absence of pain induced by management procedures


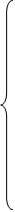
 H_1_+ (H_2_-H_1_)µ_67_ + (H_3_-H_2_)µ_7_ if H_1_ ≤H_2_ ≤H_3_
 H_1_+ (H_3_-H_1_)µ_67_ + (H_2_-H_3_)µ_6_ if H_1_ ≤H_3_ ≤H_2_
Principle for Good Health = H_2_+ (H_1_-H_2_)µ_57_ + (H_3_-H_1_)µ_7_ if H_2_ ≤H_1_ ≤H_3_ H_2_+ (H_3_-H_2_)µ_57_ + (H_1_-H_3_)µ_5_ if H_2_ ≤H_3_ ≤H_1_
 H_3_+ (H_1_-H_3_)µ_56_ + (H_2_-H_1_)µ_6_ if H_3_ ≤H_1_ ≤H_2_
 H_3_+ (H_2_-H_3_)µ_56_ + (H_1_-H_2_)µ_5_ if H_3_ ≤H_2_ ≤H_1_

μ_5_ = 0.20 μ_56_ = 0.24
μ_6_ = 0.14 μ_57_ = 0.24
μ_7_ = 0.24 μ_67_ = 0.30

*4. Behaviour*

*4.1 Expression of social behaviour*

Expression of social behaviours: B_1_ = 100 - % cows in ADF group 3 (50-100 cm).

*4.2 Expression of other behaviour*

The % days per year with at least 6 h at pasture is considered (P).

A spline function is used to compute the index P into a score with the formula:

Score for expression of other behaviours B_2_ = a + b x P+ c x P^2^ + d x P^3^

with a, b, c, d differing when P is lower or equal to a specific value (called knot) vs. equal or higher than this value.

The values for a, b, c, d and the knot are:

| knot | 50 |
| --- | --- |
| a when I < knot | 0 |
| a when I > knot | -37.3243443640749 |
| b when I < knot | 1.77560089967357 |
| b when I > knot | 4.01506156190066 |
| c when I < knot | -0.000931972606946868 |
| c when I > knot | -0.0457211858571009 |
| d when I < knot | -0.000105564708283026 |
| d when I > knot | 0.000193030046744735 |

*4.3 Human – animal relationship (ADF)*

Four categories of animals are distinguished and the % of animals in each of them are combined in a weighted sum, with the following weights:

• 0 for animals that can be touched (Avoidance Distance (AD) = 0),
• 3 for animals that can be approached closer than 50 cm but not touched (0 < AD ≤ 50),
• 11 for animals that can be approached as closely as 100 cm to 50 cm (50 < AD ≤ 100),
• 26 for animals that cannot be approached as closely as 100 cm (AD > 100).
This sum is computed into an index (I) that varies from 0 (worst situation) to 100 (best situation).

3(% cat 2) + 11(% cat 3) + 26 (%cat 4
 I= 100 - 26

A spline function is used to compute the index I into a score with the formula:

Score for good human-animal relationship B_3_ = a + b x I + c x I^2^ + d x I^3^

with a, b, c, d differing when I is lower or equal to a specific value (called knot) vs. equal or higher than this value.

The values for a, b, c, d and the knot are:

| knot | 70 |
| --- | --- |
| a when I < knot | 0 |
| a when I > knot | -261.745092092762 |
| b when I < knot | 0.738412313486647 |
| b when I > knot | 11.9499006437295 |
| c when I < knot | -0.0107770067922994 |
| c when I > knot | -0.170712864132477 |
| d when I < knot | 0.00011433123055267 |
| d when I > knot | 0.000873883669053706 |

This results in the calculation for the principle of Good Behaviour:

B_1_ = Expression of social behaviours: 100 - % cows in ADF group 3 (50-100 cm).
B_2_ = Access to OLA/Pasture
B_3_ = ADF


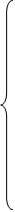
 B_1_+ (B_2_-B_1_)µ_91_ + (B_3_-B_2_)µ_10_ if B_1_ ≤B_2_ ≤B_3_
 B_1_+ (B_3_-B_1_)µ_91_ + (B_2_-B_3_)µ_09_ if B_1_ ≤B_3_ ≤B_2_
Principle for Good Behaviour = B_2_+ (B_1_-B_2_)µ_81_ + (B_3_-B_1_)µ_10_ if B_2_ ≤B_1_ ≤B_3_ B_2_+ (B_3_-B_2_)µ_81_ + (B_1_-B_3_)µ_08_ if B_2_ ≤B_3_ ≤B_1_
 B_3_+ (B_1_-B_3_)µ_89_ + (B_2_-B_1_)µ_09_ if B_3_ ≤B_1_ ≤B_2_
 B_3_+ (B_2_-B_3_)µ_89_ + (B_1_-B_2_)µ_08_ if B_3_ ≤B_2_ ≤B_1_

μ_08_ = 0.20 μ_89_ = 0.24
μ_09_ = 0.14 μ_81_ = 0.24
μ_10_ = 0.24 μ_91_ = 0.30

Final classification:

A farm is considered ‘excellent’ if it scores more than 55 on all principles and more than 80 on two of them while it is considered ‘enhanced’ if it scores more than 20 on all principles and more than 55 on two of them. Farms with ‘acceptable’ levels of animal welfare score more than 10 on all principles and more than 20 on three of them. Farms that do not reach these minimum standards are not classified.
